# *Acanthamoeba*-mediated cytopathic effect correlates with MBP and AhLBP mRNA expression

**DOI:** 10.1186/s13071-017-2547-0

**Published:** 2017-12-28

**Authors:** Sook-Luan Ng, Anisah Nordin, Norzana Abd Ghafar, Yusof Suboh, Noraina Ab Rahim, Kien-Hui Chua

**Affiliations:** 1 Department of Physiology, Faculty of Medicine, Universiti Kebangsaan Malaysia, Jalan Yaacob Latif, 56000 Kuala Lumpur, Bandar Tun Razak Malaysia; 2 Department of Parasitology and Medical Entomology, Faculty of Medicine, Universiti Kebangsaan Malaysia, Jalan Yaacob Latif, 56000 Kuala Lumpur, Bandar Tun Razak Malaysia; 3 Department of Anatomy, Faculty of Medicine, Universiti Kebangsaan Malaysia, Jalan Yaacob Latif, 56000 Kuala Lumpur, Bandar Tun Razak Malaysia

**Keywords:** Keratitis, *Acanthamoeba*, Genotype, Cytopathic, MBP, AhLBP

## Abstract

**Background:**

In recent years, the concern of *Acanthamoeba* keratitis has increased since the infection is often associated with contact lens use. Partial 18S rRNA genotypic identification of *Acanthamoeba* isolates is important to correlate with pathophysiological properties in order to evaluate the degree of virulence. This is the first report of genotypic identification for clinical isolates of *Acanthamoeba* from corneal scrapings of keratitis in Malaysia. This study is also the first to correlate the mRNA expression of MBP and AhLBP as virulent markers for axenic strains of *Acanthamoeba*.

**Results:**

In this study, ten clinical isolates were obtained from corneal scrapings. Rns genotype and intra-genotypic variation at the DF3 region of the isolates were identified. Results revealed that all clinical isolates belonged to the T4 genotype, with T4/6 (4 isolates), T4/2 (3 isolates), T4/16 (2 isolates) and one new genotype T4 sequence (T4/36), being determined. The axenic clinical isolates were cytopathogenic to rabbit corneal fibroblasts. MBP and AhLBP mRNA expression are directly correlated to *Acanthamoeba* cytopathic effect.

**Conclusions:**

All ten Malaysian clinical isolates were identified as genotype T4 which is predominantly associated with AK. Measuring the mRNA expression of *Acanthamoeba* virulent markers could be useful in the understanding of the pathogenesis of *Acanthamoeba* keratitis.

**Electronic supplementary material:**

The online version of this article (doi: 10.1186/s13071-017-2547-0) contains supplementary material, which is available to authorized users.

## Background

Photophobia, severe pain, redness and tearing are the common symptoms of *Acanthamoeba* keratitis (AK). AK is a rare but sight-threatening corneal infection which is caused by the free-living amoebae of the genus *Acanthamoeba* [1]. These organisms are widely distributed in the air, soil, water and domestic water tap, etc. [2, 3]. AK is characterized by severe pain due to radial neuritis, and a ring-like stromal infiltrate appears in the advanced stage of AK [4]. Poor vision or even visual loss occurs due to corneal scarring if proper treatment is delayed. In recent years, the concern of AK has increased since this infection is often associated with contact lens contamination, especially in urban areas. The increasing use of contact lenses for visual and cosmetic purposes, combined with improper cleaning and storage practices could be the reason for the increase in the number of AK infections [5, 6]. The first reported AK case in Malaysia was also related with a contaminated contact lens [7].

Morphological classification of *Acanthamoeba*, as described by Pussard & Pons [8] is less reliable due to the possible alteration of cyst shape by the ionic strength of the growth medium [9]. Genotyping is a useful tool in the taxonomic and epidemiological study of AK and provides a correlation between genotype and phenotypes of *Acanthamoeba* isolates [10]. Recently, the most promising method to identify the *Acanthamoeba* genotype is the sequencing of the complete nuclear 18S rRNA (Rns) gene [11]. Following this, the previous studies found that the partial 18S rRNA gene sequences which contain the *Acanthamoeba* genus-specific amplicon (ASA.S1) were sufficient to identify the *Acanthamoeba* Rns genotypes. ASA.S1 includes a region called diagnostic fragment 3 (DF3) which encodes a highly variable stem 29-1used for genotype discrimination [11, 12]. There were 20 genotypes identified and designated as T1-T4 [13], T5-T12 [14], T13 [15], T14 [16], T15 [17], T16 [18], T17 [19], T18 [20] and T19-T20 [21]. The T4 genotype is the predominant sequence type associated with AK [14]. Other Rns genotypes have also been reported as a causative agent for AK, such as T2 [22], T3 [14], T5 [10], T6 [23], T10 [19], T11 [13] and T15 [24].

Physiological properties and genotyping of *Acanthamoeba* should be studied simultaneously to evaluate the pathogenic potential of the isolates. Cytopathic tests could be used as a pathogenic marker to determine the degree of virulence of the interested *Acanthaoeba* isolates; virulent isolates are able to induce a cytopathic effect but non-pathogenic *Acanthamoeba* cannot [25]. The cytoadherence of *Acanthamoeba* to mannosylated glycoproteins on the corneal epithelial cells is a critical step to initiate the *Acanthamoeba* keratitis [26, 27]. It has been shown that the adhesion of *Acanthamoeba* to host cells is mediated by the mannose binding protein (MBP) on the surface of trophozoites. Through MBP-mediated adhesion to host cells, the amoebae produce a contact-dependent mechanism which able to exhibit a cytopathic effect involving direct cytolysis, phagocytosis, apoptosis and proteolytic activity [28].

*Acanthamoeba* trophozoites are stimulated to produce 133 kDa mannose induced protein after exposure to the upregulated mannose-specific lectins in the ulcerated corneal epithelium and lead to the activation and upregulation of matrix metalloproteinases in corneal cells [29, 30]. Besides that, the ability of *Acanthamoeba* to adhere on the laminin of the Bowman’s membrane and extracellular matrix via its *Acanthamoeba* laminin binding protein (AhLBP), is also important to allow *Acanthamoeba* to invade the corneal stroma in AK [31]. *Acanthamoeba* were capable of adhering and invading the extracellular matrix components such as laminin and collagen type I [32]. Previous studies provided information on the role of MBP and AhLBP as the marker of pathogenicity, mostly in protein level and a few in DNA works [31, 33–36].

Genotyping of *Acanthamoeba* isolates is still under-reported in Malaysia. This study, therefore, aimed to determine the Rns genotype of Malaysian clinical isolates from corneal scrapings by analyzing the intra-genotypic variation at the DF3 region. The characterization of morphological and cytopathic properties for *Acanthamoeba* isolates was studied. This study is the first evaluation of mRNA expression of MBP and AhLBP quantitatively for *Acanthamoeba* spp. and correlates both virulent markers and their growth rate with cytopathic effect.

## Methods

### Sample collection

Ten *Acanthamoeba* isolates are maintained cultures which were obtained from the *Acanthamoeba* Culture Laboratory of Parasitology Department, UKM. The source of the samples is described in Table 1. The isolates were cultured on a non-nutrient agar (NNA) plate and overlaid with 150 μl of heat-killed *Escherichia coli* at 30 °C. A small piece of agar containing cysts was removed from the primary culture and transferred to another new NNA plate, which was continued until it was totally free from co-contaminant, and the culture is known as a xenic culture. Two week-old cysts were harvested from the NNA plate and washed with PAGE amoebic saline (PAS) solution twice and then incubated overnight with 0.5 N sterile HCl during the process of axenization. The cysts were washed with PAS solution three times and centrifuged at 500× *g* for 10 min to obtain the cyst pellet. The cyst pellet was suspended with enriched PYG medium (4% peptone, 4% yeast extract, 2% glucose) supplemented with 10% fetal bovine serum (FBS; Gibco, Grand Island, New York, USA), 1% antibiotic-antimycotic (Gibco) and cultured in a T-25 flask (Greiner Bio-One, Frickenhausen, Germany) at 37 °C. The PYG medium was adjusted to pH 7.2. The axenic trophozoites were excysted in the PYG medium after 1 day of incubation. The axenic cultures were subcultured every 3 days.

**Table 1:** *Acanthamoeba* spp. isolates used in the present study for clinical isolates and reference strains

*Acanthamoeba* spp. isolates	Source	Genotype Rns/ DF3 sequence	GenBank ID
Clinical isolates			
AC20	Left corneal scraping	T4/6	KY964312
UKMAC1	Right corneal scraping	T4/6	KY964313
UKMAC2	Right corneal scraping	T4/6	KY964314
UKMAC3	Right corneal scraping	T4/6	KY964315
UKMAC4	Right corneal scraping	T4/22	KY964316
UKMAC5	Right corneal scraping	T4/2	KY964317
UKMAC6	Left corneal scraping	T4/16	KY964318
UKMAC7	–	T4/2	KY964319
UKMAC8	Corneal scraping	T4/2	KY964320
UKMAC9	Left corneal scraping	T4/16	KY964321
Reference strains			
P1CS	Corneal scraping	T4/1	AF441812
P91CS	Corneal scraping	T4/2	AF441808
P97LCSS1	Contact lens case solution	T4/3	AF441794
P97RCLS2	Right contact lens	T4/4	AF441796
P97LCLS2	Left contact lens	T4/5	AF441795
P120CS	Corneal scraping	T4/6	AF441810
P209CS	Corneal scraping	T4/7	AF441802
C10TA	Tape	T4/8	AF441799
C68TA	Tape	T4/9	AF441803
C124LC	Contact lens case	T4/10	AF441798
BP:P20:LCS	Left corneal scraping	T4/11	FJ422511
BP:P8:LCS	Left corneal scraping	T4/12	FJ422512
BP:P1:RCS	Right corneal scraping	T4/13	FJ422513
BP:P6:LCS	Left corneal scraping	T4/14	FJ422524
BP:P7:RCL	Right corneal scraping	T4/15	FJ422526
BP:P15:RCS	Right corneal scraping	T4/16	FJ422527
BP:P9:LCS	Left corneal scraping	T4/17	FJ422537
BP:P14:LCS	Left corneal scraping	T4/18	FJ422533
BP:P22:LCS	Left corneal scraping	T4/19	FJ422535
BP:P13:CB	Cornea	T4/20	FJ422536
BP:P16:RCS	Right corneal scraping	T4/21	FJ422541
AKSI001	Corneal scraping	T4/22	GQ342612
AKSI002	Corneal button	T4/23	GQ342613
AKSI003	Corneal button	T4/24	GQ342614
AKSI004	Corneal button	T4/25	GQ342615
AKSI008	Corneal button	T4/26	GQ342619
AKSI011	Corneal button	T4/27	GQ342622
AKSI014	Corneal button	T4/28	GQ342625
AC6 (T4/22)	Corneal scraping	T4/29	AB554223
AC15 (T4/23)	Corneal scraping	T4/30	AB554225
CDC V328	GAE, brain	T4/31	AY702999
AcL-JN15	Corneal scraping	T4/32	HF930505
AcL-LA16	Keratitis	T4/33	HF930509
AcL-GF8	Corneal scraping	T4/34	HF930500
WAL (T4/29)	Keratitis	T4/35	JX441875
*A. castellanii* CDC:0981:V006	GAE, brain, Georgia, USA	T1	U07400
*A. palestinensis* Reich ATCC30870	Soil, Israel	T2	U07411
*A. griffini* (H37)	Keratitis, Scotland, UK	T3	S81337
*A. griffini* S-7 ATCC30731	Beach bottom, Connecticut, USA	T3	U07412
*A. culbertsoni* Diamond	Keratitis, Ohio, USA	T4	AF019057
*A. castellanii* CDC:0814:V014	Keratitis, India	T4	U07401
*A. castellanii* Castellani ATCC50374	Yeast culture, UK	T4	U07413
*A. polyphaga* ATCC30461	Human corneal scraping, Houston, Texas, USA	T4	AY026243
*Acanthamoeba* sp. ATCC 50369	Keratitis, Texas, USA	T4	U07409
*A. hatchetti* strain 2HH	Keratitis, Austria	T4	AF260722
*A. lenticulata* Jc-1 ATCC50428	Fresh water stream, New York, USA	T5	U94739
*A. lenticulata* strain 45 ATCC50703	Human nasal mucosa, Germany	T5	U94730
*A.lenticulata* PD2S ATCC30841	Swimming pool, France	T5	U94741
*A. palestinensis* 2802 ATCC50708	Swimming pool, France	T6	AF019063
*A. astronyxis* Ray Hayes ATCC30137	Laboratory water, Washington, USA	T7	AF019064
*A. tubiashi* OC-15C ATCC30867	Fresh water, Maryland, USA	T8	AF019065
*A. comandoni* ATCC30135	Soil, France	T9	AF019066
*A. culbertsoni* Lily A-1 ATCC30171	Human cell culture, Indiana, USA	T10	AF019067
*A. hatchetti* BH-2	Brackish water, Maryland, USA	T11	AF019068
*A. healyi* CDC 1283:V013	GAE, brain, Barbados, BWI	T12	AF019070
*Acanthamoeba* sp. UWET39	Soil, Washington, USA	T13	AF132136
*Acanthamoeba* sp. UWC9	Contact lens case	T13	AF132134
*Acanthamoeba* sp. PN15	Clinical sample, Pakistan	T14	AF333607
*A. jacobsi* AC005 ATCC30732	Sea sediment, New York Bight Apex	T15	AY262360
*A. jacobsi* AC305	Untreated water system, Australia	T15	AY262365
*Acanthamoeba* sp. cvX	Freshwater pond, Italy	T16	GQ380408
*Acanthamoeba* sp. U/H-C1	Freshwater pond, Italy	T16	AY026245
*Acanthamoeba* sp. Ac E1a	Lampangpuri pond, Bangkok, Thailand	T17	GU808277
*Acanthamoeba* sp. Ac E9b	Freshwater pond, National Stadium, Bangkok, Thailand	T17	GU808302
*Acanthamoeba* sp. CDC: V621 clone 10	GAE	T18	KC822470
*Acanthamoeba* sp. USP AWW A68	Water treatment plant, Spain	T19	KJ413084
*Acanthamoeba* sp. AM-3H/T16	River water, Poland	T20	HQ632777
*Acanthamoeba* sp. M22/T16	Bronchoaspirate fluid, Poland	T20	GQ342607
*Acanthamoeba* sp. OSU 04–023 clone 2	Liver tissue, Toucan	T20	DQ451162
*Acanthamoeba* sp. OSU 04–020 clone 2	Liver tissue, Toucan	T20	DQ451161

### Identification of *Acanthamoeba*

A wet smear of xenic cultures was observed under a light microscope for the morphology features of trophozoites and cysts. The diameter of cysts was measured using the VideoTest Morphology software (VideoTest, Saint Petersburg, Russia, version 5). Isolates were classified as *Acanthamoeba* spp. groups I, II or III according to the keys of Pussard & Pons [8].

The 18S rDNA identification of *Acanthamoeba* spp. was performed as per the following procedures. *Acanthamoeba* isolates with a density of 5 × 10^5^ trophozoites were harvested from NNA plates and DNA was extracted with a commercially available AccuPrep® Genomic DNA Extraction Kit (Bioneer, Daejeon, Korea). The pelleted trophozoites were resuspended in 200 μl PAS solution, mixed with 20 μl proteinase K and 200 μl binding buffer and incubated at 60 °C for 10 min. The lysate was transferred into the upper reservoir of the binding column tube after added 100 μl isopropanol. The tube was centrifuged at 8000× *g* for 1 min. The binding column tube was washed with 500 μl ethanol twice and the DNA was eluted with 200 μl elution buffer. After that, amplicon ASA.S1 was amplified by PCR using Platinum® Taq DNA Polymerase High Fidelity kit (Invitrogen, Carlsbad, CA, USA) following the recommended protocol with genus-specific primers JDP1 and JDP2 (Table 2). The PCR reaction was started with incubation at 94 °C for 3 min, followed with 35 cycles of 30 s at 94 °C, 30 s at 61 °C and 1 min at 72 °C. Incubation for another 5 min at 72 °C was done for final extension. Amplification products were visualized by ethidium bromide staining in 1.5% agarose gel electrophoresis and followed by gel extraction using PureLink™ quick gel extraction kit (Invitrogen). ASA.S1 PCR products were ligated into a plasmid vector by using a T/A cloning kit (Invitrogen). Plasmids of identified positive clones were extracted using PureLink™ Quick Plasmid Miniprep kit (Invitrogen) and sequenced with ABI prism™ Bigdye™ terminator cycle using primer M13 (Applied Biosystems, Foster City, CA, USA).

**Table 2:** Primers used for PCR and RT-PCR reactions in this study

Accession no.	Gene	Direction	Sequence	Amplicon size (bp)	Source
Genotyping primers					
JDP	ASA.1	Forward	5′-GGCCCAGATCGTTTACCGTGAA-3′	423–551	[11]
		Reverse	5′-TCTCACAAGCTGCTAGGGAGTCA-3′		
*Acanthamoeba* virulent marker primers					
U29609	ARP2	Forward	5′-GCTGTCTTGACCCTCTACGC-3′	101	Present study
		Reverse	5′-AGCGAGAAGCCCTCGTACAC-3′		
AY604040	MBP	Forward	5′-AGGGCGAGACCTACGATAGC-3′	165	Present study
		Reverse	5′-CCTCGTAGACGAAGGTGAGG-3′		
AY351649	AhLBP	Forward	5′-CCAACACCGACTCTCCTCTC-3′	183	Present study
		Reverse	5′-CTCCTCAGGGTCACGGTAGA-3′		

The alignment of interesting sequences with homologous sequences from GenBank was conducted using BLAST (http://www.ncbi.nlm.nih.gov). Phylogenetic analyses were conducted in MEGA7 (Molecular Evolutionary Genetic Analysis software, version 7) [37]. The evolutionary history was inferred using the Neighbor-Joining method [38]. The bootstrap consensus tree inferred from 1000 replicates [39] was taken to represent the evolutionary history of the taxa analyzed [39]. Branches corresponding to partitions reproduced in fewer than 50% bootstrap replicates were collapsed. The percentage of replicate trees in which the associated taxa clustered together in the bootstrap test (1000 replicates) was shown next to the branches [39]. The tree was drawn to scale, with branch lengths in the same units as those of the evolutionary distances used to infer the phylogenetic tree. The evolutionary distances were computed using the Kimura 2-parameter method [40] and were in the units of the number of base substitutions per site. All positions containing alignment gaps and missing data were eliminated only in pairwise sequence comparisons. The alignment of DF3 sequences was performed using MEGA 7 with ClustalW. The reference sequences used in this study are available in GenBank under the accession numbers shown in Table 1.

### Cytopathic effect

Rabbit corneal fibroblasts were isolated from the cornea of New Zealand white strain rabbits as described in the previous study [41]. The rabbit heads were bought from the slaughter farm in Ijok, Malaysia. The corneal stroma was digested using collagenase type I solution in order to isolate the corneal fibroblasts. The corneal fibroblasts were then cultured on a 24 × 24 mm coverslip in 35 mm tissue culture dishes (Orange Scientific, Braine-L’Alleud, Belgium) with F12:DMEM supplemented with 10% FBS, 1% Glutamax, 1% antibiotic-antimycotic (Invitrogen) at 37 °C with atmospheric O^2^ and 5% CO^2^ level. The cell culture medium contained sodium bicarbonate and HEPES solution which was used as buffer to maintain the media at pH 7.2-7.6. The 100% confluent cells were treated with 3 densities of axenic trophozoites, which were 10^4^, 10^5^ and 10^6^ trophozoites per dish/well for triplicates at 3 time intervals (3, 6 and 24 h). The cells were fixed using 4% paraformaldehye and stained with giemsa. The area of remaining cells after cytolysis was observed under a light microscope and measured using the VideoTest Morphology software. The percentage of cytopathic effect (CPE) was calculated based on the surface area of empty spaces in the culture.

### Growth rate of trophozoites

The growth rate of axenic trophozoites was performed in triplicates based on the trypan blue exclusion method using a haemocytometer (Weber Scientific International Ltd., Teddington, UK). Each axenic isolate was seeded with 1 × 10^5^ trophozoites per well into 6-well plate (Nunc™, Wiesbaden, Denmark) and cultured with PYG medium at 37 °C for 24 h. The harvested trophozoites were stained with trypan blue vital dye (Gibco) and then directly counted. The formula for the growth rate was: difference between the final live trophozoite count and initial seeding, divided by the surface area (9.6 cm^2^) and days of confluence (1 day). Each sample with 1 million axenic trophozoites was lysed using 1 ml TRI reagent in order to preserve the total RNA.

### mRNA expression of *Acanthamoeba* mannose binding protein and laminin binding protein

Total RNA extraction of axenic *Acanthamoeba* was performed using TRI Reagent (Molecular Research Center, Cincinnati, USA) according to the manufacturer’s protocol. The lysate was then separated into the aqueous and organic phases with the addition of 200 μl chloroform and centrifugation at 12,000× *rpm* for 15 min. RNA remained exclusively in the aqueous phase and precipitated by 500 μl isopropanol and 5 μl polyacryl carrier (Molecular Research Center). The precipitated RNA was washed with 1 ml 75% ethanol and solubilized with 20 μl RNAse and DNAse free distilled water (Invitrogen). The quantity and purification of total RNA were determined by a Nanodrop ND-100 spectrophotometer (Nanodrop Technologies, Wilmington, Delaware, USA). SuperScript™ III First-Strand Synthesis SuperMix (Invitrogen) was used to synthesise complementary DNA (cDNA) from 100 ng of total RNA according to the manufacturer’s protocol. The master mix of cDNA synthesis included 10 μl of 2× reaction mix, 5 μl total RNA, 3 μl diethylpyrocarbonate-treated water and 2 μl reverse transcriptase enzyme. The reverse transcription was initiated with 10 min at 23 °C for primer annealing, 60 min at 50 °C for reverse transcription and 5 min at 85 °C for reaction termination. The primers (forward and reverse) were designed from NIH GenBank using Primer Output 3 software (Table 2). *Acanthamoeba* actin related protein 2 (ARP2) is used as housekeeping gene in this study [42, 43]. Quantitative polymerase chain reactions were performed using MyiQ cycler (Bio-Rad, Hercules, CA, USA) with iQ™ SYBR® Green Supermix (Bio-Rad, Hercules, CA, USA). The master mix of qRT-PCR consisted of 12.5 μl of 2× SYBR Green Supermix, 1 μl cDNA, 9.5 μl RNase and DNase free distilled water, 1 μl of 5 μM forward primer and 1 μl of 5 μM reverse primer. The reaction profile involved pre-denaturation for 3 min at 95 °C, PCR amplification for 40 cycles of 30 s at 95 °C and 30 s at 61 °C, with a final extension for 1 min at 72 °C and melting curve analysis. The specificity of PCR products was confirmed with melting curve analysis and 1.5% agarose gel electrophoresis. Relative mRNA expression of the virulent genes was calculated based on their qRT-PCR threshold cycle (Ct) value using the formula of 2^(housekeeping gene Ct value - virulent gene Ct value).

### Statistical tests

Quantitative data were tested for statistical significance using the Statistical Package for Social Sciences version 20 (IBM Corporation, Armonk, New York, USA). Student’s t-test for independent samples was performed for normally distributed data, while the Mann-Whitney U-test was used as a non-parametric test. Correlation between two variables was analysed using Pearson’s correlation coefficient. The results were presented as the mean ± standard error mean (SEM). *P* < 0.05 was considered to be statistically significant.

## Results

### Identification of *Acanthamoeba*

**Fig. 1:**
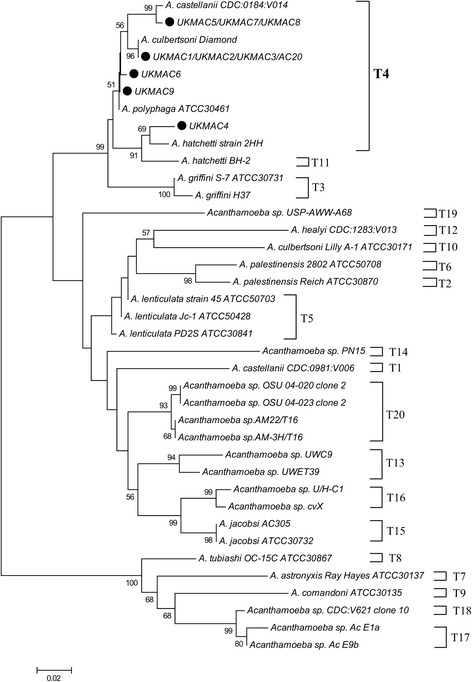
Evolutionary relationships of taxa. The evolutionary history was inferred using the neighbor-joining method for the 18S rDNA partial sequences of clinical isolates (black dots). The optimal tree with the sum of branch length = 1.25072993 is shown. The percentage of replicate trees in which the associated taxa clustered together in the bootstrap test (1000 replicates) is shown next to the branches. The tree is drawn to scale, with branch lengths in the same units as those of the evolutionary distances used to infer the phylogenetic tree. The evolutionary distances were computed using the Kimura 2-parameter method and are in the units of the number of base substitutions per site. The analysis involved 38 nucleotide sequences. There were a total of 309 positions in the final dataset. Evolutionary analyses were conducted with MEGA7

**Fig. 2:**
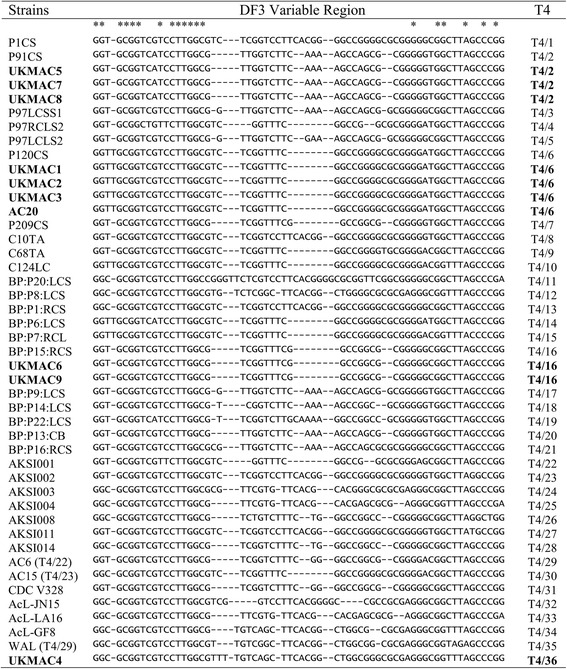
T4 subgenotype of clinical isolates. Primary sequence alignment of a subset of the highly variable and informative region of DF3 (stem 29-1, 18S rRNA) of Malaysian clinical isolates from corneal scrapings and the reference sequences of the T4 Rns genotype. Abbreviations for samples and reference sequences are as defined in Table 1. Sequences were aligned by similarity. Asterisks denote similar positions and gaps are represented as dashes. Samples of the present study are indicated in bold

In this study, all the Malaysian clinical isolates from corneal scrapings were identified as Rns genotype T4. Currently, there were 35 T4 subgenotypes with deposited GenBank data and published in the articles, which were T4/1 to T4/10 [12], T4/11 to T4/21 [10] and T4/22 to T4/28 [44]. Another study also independently reported the same designation of T4/22 and T4/23 on the same year [45]. Therefore, Risler et al. [46] renamed the designation of T4/22 and T4/23 identified by the study of Abe & Kimata [45] to T4/29 and T4/30. Besides that, the T4 subgenotypes from T4/31 to T4/34 were also reported by them [46]. On the same year, another study also reported the same designation to T4/29 independently [47]. Thus, this study named the T4/29 identified by Duarte et al. [47] to T4/35. We found that isolate UKMAC4 possessed a new T4 sequence in the GenBank database. This isolate was 98% similar to *A. hatchetti* strain 2HH from a keratitis patient in Austria. Hence, isolate UKMAC4 was designated as T4/36 in this study. On the other hand, 8 out of 10 isolates have been determined phylogenetically as identical to other previously identified genotype T4 Rns isolates. The sequences of UKMAC1, UKMAC2, UKMAC3 and AC20 were similar to *A. culbertsoni* Diamond and belonged to T4/6 based on the DF3 sequence determination. Rns sequencing also demonstrated that three isolates, UKMAC5, UKMAC7 and UKMAC8 were identical to each other and similar to two ocular samples from France, which were *Acanthamoeba* spp. S22 and S36 (DQ87314 and EU146073, respectively). These isolates were determined as T4/2 and also 99% similar to *A. castellanii* CDC:0184:V014. Both samples of UKMAC6 and UKMAC9 with DF3 sequence T4/16 were isolated from the patient’s left corneal scrape. UKMAC9 was identical to *A. polyphaga* ATCC30461, while UKMAC6 was 99% similar to UKMAC9 where only one nucleotide substitution was found in the outer region of the DF3 sequence. The genotyping results are summarized in Table 1 and Table 3 and the phylogenetic tree is shown in Fig. 1. The T4 subgenotyping result is shown in Fig. 2. All sequences generated in this study were submitted to the GenBank database under the accession numbers KY964312–KY964321 (Table 1).

**Table 3:** Morphological group and cyst diameter for the clinical isolates and percentage of sequence similarity with homologous sequences

*Acanthamoeba* isolate	Group	Cyst diameter ± SEM (μm)	BLAST result similarity to homologous sequence (%)
UKMAC1	III	15.21 ± 0.17	*A. culbertsoni* Diamond (100%)
UKMAC2	III	15.13 ± 0.30	*A. culbertsoni* Diamond (100%)
UKMAC3	III	15.07 ± 0.24	*A. culbertsoni* Diamond (100%)
AC20	III	13.43 ± 0.15	*A. culbertsoni* Diamond (100%)
UKMAC5	II	16.54 ± 0.17	*A. castellanii* CDC:0184:V014 (99%)
UKMAC7	II	16.55 ± 0.19	*A. castellanii* CDC:0184:V014 (99%)
UKMAC8	II	16.46 ± 0.09	*A. castellanii* CDC:0184:V014 (99%)
UKMAC9	II	17.20 ± 0.23	*A. polyphaga strain* ATCC30461 (100%)
UKMAC6 (~ 99% UKMAC9)	II	16.73 ± 0.52	*A. polyphaga strain* ATCC30461 (99%)
UKMAC4	II	16.29 ± 0.27	*A. hatchetti strain* 2HH (98%)

**Fig. 3:**
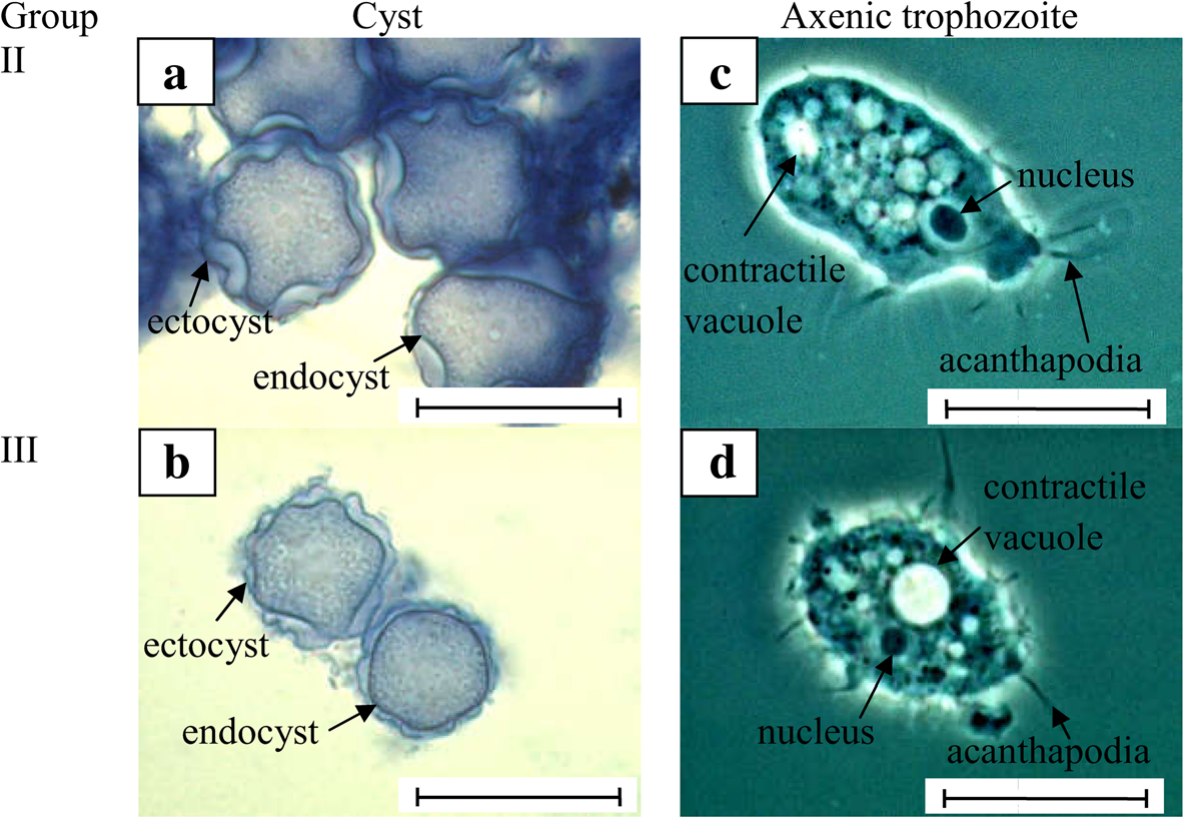
Morphology of cysts and axenic trophozoites of group II and III *Acanthamoeba* stained with methylene blue and observed under phase contrast microscopy (magnification ×1000). **a** Thick ectocyst and polygonal endocyst in Group II. **b** Thin ectocyst adjacent to the endocyst in Group III. **c**, **d** *Acanthamoeba* trophozoites with characteristic acanthapodia, contractile vacuole and prominent nucleus. *Scale-bars*: 20 μm

Based on Pussard & Pons’ [8] morphological classification, the result indicated that only four isolates (UKMAC1, UKMAC2, UKMAC3 and AC20) belonged to Group III with the cyst size < 18 μm and a smooth endocyst. The range of cyst diameters are represented in Table 3. Another six isolates belonged to Group II, where the cyst sizes were < 18 μm and a polygonal endocyst with 6–7 arms (Fig. 3 and Table 3). Xenic samples were able to excyst and proliferate robustly at 37 °C. Only five isolates (AC20, UKMAC2, UKMAC4, UKMAC7 and UKMAC8) were able to be axenized and grown well in the liquid medium at human body temperature (37 °C).

### Cytopathic effect

**Fig. 4:**
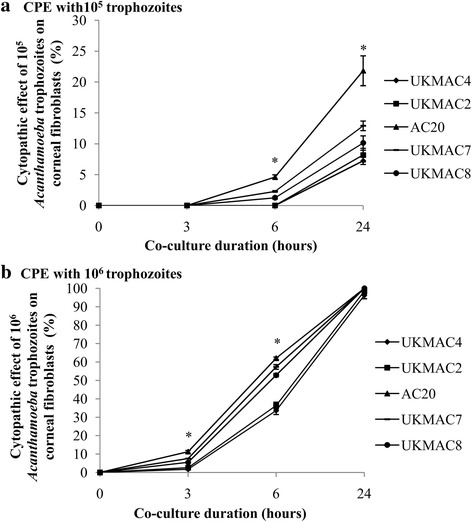
Cytopathic effect of *Acanthamoeba* trophozoites on corneal fibroblasts. The seeding density of *Acanthamoeba* was 10^5^ (**a**) and 10^6^ trophozoites (**b**) for 3, 6 and 24 h co-culture. AC20 had a significantly higher CPE than all other isolates (*P < 0.05)

**Fig. 5:**
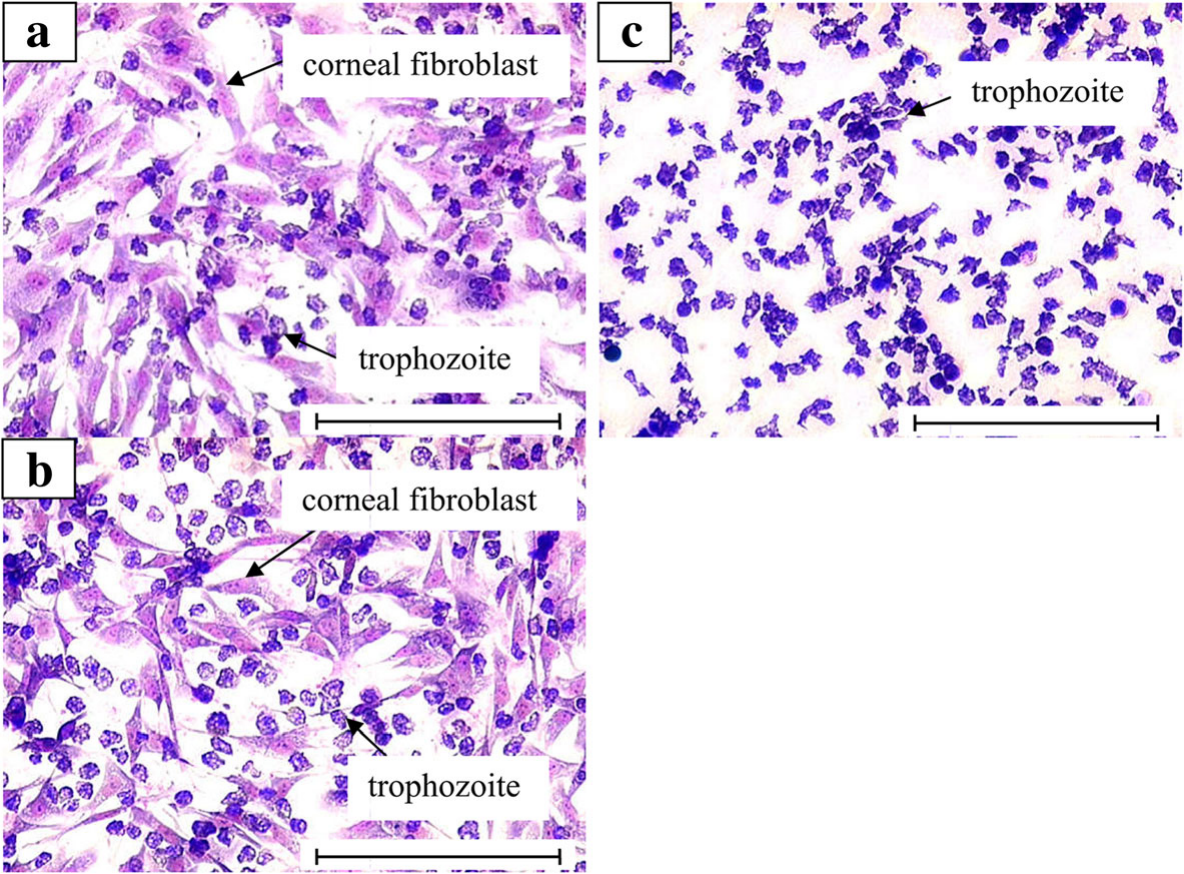
Cytopathic effect of AC20 isolate on the corneal fibroblasts with seeding of 10^6^ trophozoites. **a** The corneal fibroblasts are fusiform; the *Acanthamoeba* trophozoites with prominent contractile vacuoles were feeding on the edge of cells and thus forming multiple small lesions on the monolayer cells after 3 h co-culture. **b** The gaps between keratocytes were increased with the time due to the cytopathic effect of trophozoites after 6 h co-culture. **c** All corneal fibroblasts were lysed by trophozoites after 24 h co-culture and only left trophozoites in the vessel. Giemsa staining (magnification ×100). *Scale-bars*: 200 μm

Cytopathic effect of 5 axenic isolates was carried out for three time intervals (3, 6 and 24 h) with three different densities of trophozoite (10^4^, 10^5^ and 10^6^ trophozoites/well) on corneal fibroblasts as shown in Fig. 4. There was no cytopathic effect been observed after 24 h co-incubation with the density of 10^4^ trophozoites/well. The cytopathic effect of corneal fibroblasts was observed after 6 h co-incubation with 10^5^ trophozoites and 3 h co-incubation with 10^6^ trophozoites. Sample AC20 exhibited the highest CPE percentage in both densities of 10^5^ and 10^6^ trophozoites/well throughout the three time intervals. The trophozoites accumulated at the edge of corneal fibroblasts and multiple small lesions were formed due to its cytopathic effect (Fig. 5). The empty spaces between the corneal fibroblasts were increased due to the cytolysis. The corneal fibroblasts had fully been lysed (100%) after 24 h co-incubation with 10^6^ trophozoites. The cells were replaced by trophozoites and a few cysts which were either attached on the well surface or floating in the medium.

### Cytopathic effect correlates with MBP and AhLBP gene expression

**Fig. 6:**
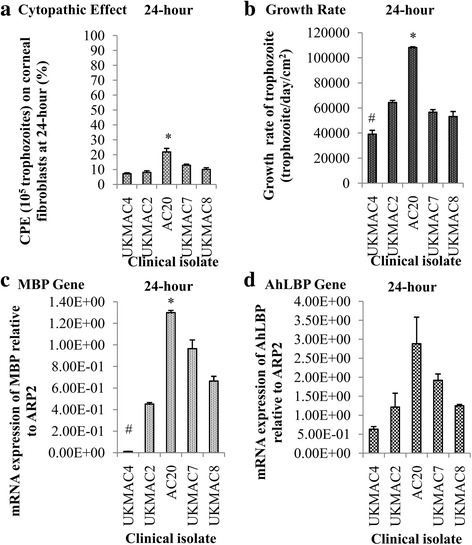
Cytopathic effect (**a**), (**b**) growth rate, mRNA expression of MBP (**c**) and AhLBP (**d**) virulent markers of clinical isolates at 24 h. AC20 had a statistically significant higher CPE, growth rate and MBP expression as compared to the other isolates (^*^ *P* < 0.05). UKMAC4 had a statistically significant lower growth rate and MBP expression as compared to the other isolates (^#^ *P* < 0.05)

**Fig. 7:**
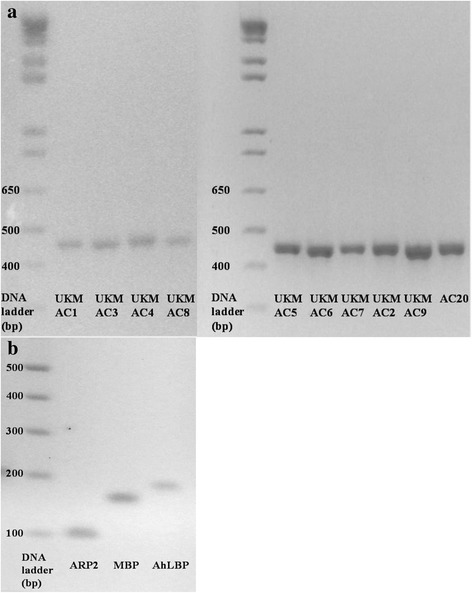
Agrose gel electrophoresis of PCR products. The products were for the target gene ASA.1 for ten *Acanthamoeba* isolates (**a**) and the housekeeping gene ARP2, *Acanthamoeba* virulent markers MBP and AhLBP genes (**b**). A single band of PCR product indicated the specificity of the synthesised primers

Sample AC20 is the most virulent strain in this study due to it having the highest trophozoite growth rate, gene expression of MBP, and cytopathic effect (Fig. 6). In comparison, the growth rate of trophozoites and gene expression of MBP were the lowest for the strain UKMAC4 among 4 axenic isolates. This study showed a positive correlation between growth rate and cytopathic effect, *r*_(15)_ = 0.889, *P* < 0.0001. There was a positive relationship between MBP gene expression and cytopathic effect, *r*_(15)_ = 0.864, *P* < 0.0001. There was also a strong relationship between AhLBP gene expression and cytopathic effect, *r*_(15)_ = 0.934, *P* < 0.0001. Specific primers of the studied genes produced a single melt peak in melting curve analysis (Additional file 1: Fig. S1) and a single band of PCR product in 1.5% agarose gel electrophoresis (Fig. 7). The statistical results of growth rate, virulent gene expression and CPE are shown in Additional file 2: Table S1.

## Discussion

Rns genotypic identification for *Acanthamoeba* is important in studying the epidemiology of *Acanthamoeba* keratitis, providing the correlation between genotype and infection [10]. Previous studies found that the T4 genotype is the most predominant causative agent for AK and also morphologically belonging to Group II and III [13]. In this study, all clinical isolates from corneal scrapings were also assigned to genotype T4, thus providing evidence to support the genotype T4 as a dominant sequence associated with AK in Malaysia. Based on DF3 sequence determination, four isolates (T4/6), three isolates (T4/2), two isolates (T4/16), and one new genotype T4 sequence were identified. The new genotype T4 was suggested as a subgenotype T4/36 in this study. Results revealed that genotype T4/2 and T4/6 were common in keratitis isolates in Malaysia and similar results had been reported from Hong Kong [12], North China [48], France [49] and the USA [10]. Our studied isolates were mainly phylogenetically identical to *A. culbertsoni* Diamond [14]. From the previous study, 4 out of 11 *Acanthamoeba* isolates (19.05%) from air-conditioners in Kuala Lumpur, Malaysia were genotypically identical to *A. culbertsoni* Diamond from keratitis, Ohio, USA [50]. This explained that the distribution of *Acanthamoeba* with a specific genotype also plays a vital rule in the occurrence of AK. T4 isolates are more prevalent and geographically widespread across the world and this dominant sequence is mostly associated with human infection [14, 24, 51].

A significant issue with the use of contact lenses is that of corneal ulcers, arising from the prolonged wearing of the lens. Contaminated contact lenses or exposure to *Acanthamoeba* accidentally from the environment could be a risk factor for AK. *Acanthamoeba* can easily invade the cornea in the event of an ulcer or trauma because mannose glycoprotein would be upregulated in response to the corneal abrasion, and thus increasing the affinity of *Acanthamoeba* trophozoites in adhering to the corneal epithelium [30]. This reveals that improper contact lens cleaning practices is most likely to be the reason of AK infection [52]. Contact lenses are a good vehicle to transport the *Acanthamoeba* to the cornea. The biofilm of contact lenses and carbohydrate residues secreted by host could be a good breeding ground and food source for *Acanthamoeba* to multiply and attach to the contact lens [53]. Moreover, wearing contaminated contact lenses increases the exposure period of *Acanthamoeba* on the cornea, thus increasing the possibility of AK, especially for those with corneal abrasion or injury.

The density of trophozoites is highly important in causing a cytopathic effect on the monolayer cells in vitro. No cell damage was observed when small inoculums of amoeba were used in the cytopathic effect test [54, 55]. In this study, 10^4^ trophozoites failed to cause a cytopathic effect even when the incubation time was prolonged to 1 week. The cut off point for corneal fibroblasts is the seeding density of 10^5^ trophozoites needed to demonstrate the cytolysis. This revealed that other than the susceptibility of *Acanthamoeba* to binding with corneal receptors, the ability to multiply and adapt in the cornea is also important in the development of AK. The sequential events of CPE were demonstrated as similar to the previous studies [55, 56]. Cell shrinkage and gaps were observed at the initial phase of CPE, then some cells were rounded and either engulfed by trophozoites or floating off. The trophozoite-mediated cytopathic effect is due to three independent mechanisms: direct cytolysis, phagocytosis and apoptosis [57]. The clinical strain of *Acanthamoeba* induced a cytopathic effect on human corneal fibroblasts mainly via apoptosis after direct adhesion rather than through soluble factors [58]. The cytopathic effect was in a dose-dependent manner after co-culture with *Acanthamoeba*, T4 *Acanthamoeba* isolates from AK patients and exhibited a similar cytopathic effect on human corneal fibroblasts [58].

In this study, the pathogenic potential of parasites in vitro is directly correlated with the expression level of the MBP. Pathogenic strains of *Acanthamoeba*, which produce robust amounts of MBP, bind to host cells and produce amoeba-induced cytopathic effects in a mannose-dependent manner. In contrast, non-pathogenic strains, which produce little or no MBP, are unable to bind onto the host cells or produce CPE [27, 28]. The ability of parasites to bind to host cells and produce CPE is directly correlated with the expression of the MBP. *Acanthamoeba* strains that expressed high level of MBP could bind strongly to host cells and produce potent CPE. The production of MBP was detected by affinity chromatography and western blot analyses [33]. The level of AhLBP mRNA expression was compared between the pathogenic and non-pathogenic *Acanthamoeba* [31]. Highly virulent strains expressed a higher level of AhLBP mRNA through northern blot analysis. The pathogenic strain has a higher affinity to attach on the extracellular matrix glycoprotein laminin when compared to the non-pathogenic strain in order to induce CPE [26].

Previous studies proved that the mannose-binding proteins and laminin-binding proteins on *Acanthamoeba* are the virulence proteins responsible for the pathogenesis of *Acanthamoeba* infection [26, 31, 35, 59]. Pre-treated amoeba with the mannose showed a significant decrease in their adhesion and invasion on the collagen matrix and reduced their cytopathic effect [32, 36]. Incubation amoeba with mannose sugars inhibited their attachment on inert surfaces in a dose-dependent way [59, 60]. *Acanthamoeba* trophozoites pretreated with anti-MBP IgY antibody significantly inhibited the CPE. Pathogenic *A. culbertsoni* exhibited high attachment on laminin and cytopathic effect on normal human keratocytes regardless of incubation with or without laminin [26].

This study showed strong a correlation between virulent genes and growth rate. Virulent strains have a higher growth rate and as well as higher virulent gene expression. Physiological characteristics correspond to their degree of virulence. It is also plausible that more trophozoites will cause more cytopathic effect. Pathogenic potential usually correlates to a high growth rate of amoeba [61], although the rapid growth of amoeba on the cells was not necessarily comparable to their growth rate on the axenic culture [62]. Cytopathic potential per trophozoite is more important in affecting the degree of cytopathic effect when compared to their numbers [63].

Although samples UKMAC2 and AC20 have the same DF3, they have a different cytopathic effect, growth rate and expression level of virulent genes. DF3 was used to differentiate their genotype but did not reveal the same phenotype in this study. The difference of phenotypes could be a consequence of developmental variation [64]. Moreover, a complete sequence may be needed to study a relation between the sequence type and phenotypes. Previous studies showed that the sequences belonging to the same DF3 could be with a different V4 region [46]. The V5 variable region includes the highly variable DF3 region and the V4 region located within the genotypic extended fragment were examined to differentiate the *Acanthamoeba* genotypes [46, 65].

## Conclusions

This study is the first report of 18S rDNA identification for clinical isolates in Malaysia. All pathogenic strains belonged to genotype T4 and one new subgenotype T4/36 (UKMAC4) was identified. This study also showed the first correlation between mRNA expression of *Acanthamoeba* virulent markers MBP and AhLBP with cytopathic effect. The expression of virulent markers is directly correlated with the cytopathic effect. The detection of mRNA expression of genes MBP and AhLBP can be used to determine the pathogenicity of *Acanthamoeba* and to study the pathogenesis of *Acanthamoeba* keratitis.

## Additional files

Additional file 1: Figure S1. Melt curve and melt peak of genes ARP2, MBP and AhLBP in qRT-PCR. **a** The melting points of genes ARP2, MBP and AhLBP were 86.5 °C, 86.5 °C and 87.5 °C, respectively. **b** Specific primers of the studied genes produced a single melt peak for each PCR product. (TIFF 6503 kb)

Additional file 2: Table S1. Statistically significant results of cytopathic effect, trophozoite growth rate and virulent gene expression. The Student’s t-test was performed for normally distributed data, while the Mann-Whitney U- test was used as non-parametric test. (DOCX 15 kb)

## Abbreviations

AhLBP: *Acanthamoeba* laminin binding protein
AK: *Acanthamoeba* keratitis
ARP2: *Acanthamoeba* actin related protein 2
ASA.S1: *Acanthamoeba* genus-specific amplicon
cDNA: complementary DNA
CPE: cytopathic effect
Ct: threshold cycle
DF3: diagnostic fragment 3
MBP: mannose binding protein
NNA: non-nutrient agar
PAS: PAGE amoebic saline
PYG: peptone, yeast extract, glucose
Rns: 18S rRNA
SEM: standard error of the mean
