# Supplementary material for: Acanthamoeba-mediated cytopathic effect correlates with MBP and AhLBP mRNA expression
Source: Parasit Vectors. 2017 Dec 28;10:625. doi: 10.1186/s13071-017-2547-0 (PMC5745754; doi:10.1186/s13071-017-2547-0)
Supplement: Supplementary file 2 — Statistically significant results of cytopathic effect, trophozoite growth rate and virulent gene expression. The Student’s t-test was performed for normally distributed data, while the Mann-Whitney U- test was used as non-parametric test. (DOCX 15 kb) [file 13071_2017_2547_MOESM2_ESM.docx]

**Additional file 2: Table S1** Significant statistical results of cytopathic effect, trophozoite’s growth rate and virulent gene expression.

| Isolate | Isolate | Statistical test | Statistical results |
| --- | --- | --- | --- |
| (a) Cytopathic effect (10^5^ trophozoites; 6-hour co-incubation) | | | |
| AC20 | UKMAC7 | Student’s T | T(4) = 5.855, *P* = 0.004 |
|  | UKMAC8 | Student’s T | T(4) = 7.747, *P* = 0.001 |
| UKMAC7 | UKMAC8 | Student’s T | T(4) = 4.060, *P* = 0.015 |
| (b) Cytopathic effect (10^5^ trophozoites; 24-hour co-incubation) | | | |
| UKMAC4 | AC20 | Student’s T | T(4) = 5.873, *P* = 0.004 |
|  | UKMAC7 | Student’s T | T(4) = 5.824, *P* = 0.004 |
| UKMAC1 | AC20 | Student’s T | T(4) = 5.156, *P* = 0.007 |
|  | UKMAC7 | Student’s T | T(4) = 3.539, *P* = 0.024 |
| AC20 | UKMAC7 | Student’s T | T(4) = 3.518, *P* = 0.025 |
|  | UKMAC8 | Student’s T | T(4) = 4.369, *P* = 0.012 |
| (c) Cytopathic effect (10^6^ trophozoites; 3-hour co-incubation) | | | |
| UKMAC4 | UKMAC1 | Student’s T | T(4) = 6.934, *P* = 0.002 |
|  | AC20 | Student’s T | T(4) = 12.494, *P* < 0.0001 |
|  | UKMAC7 | Student’s T | T(4) = 44.678, *P* < 0.0001 |
|  | UKMAC8 | Student’s T | T(4) = 9.523, *P* < 0.0001 |
| UKMAC1 | AC20 | Student’s T | T(4) = 11.298, *P* < 0.0001 |
|  | UKMAC7 | Student’s T | T(4) = 31.312, *P* < 0.0001 |
|  | UKMAC8 | Student’s T | T(4) = 7.205, *P* = 0.002 |
| AC20 | UKMAC7 | Student’s T | T(4) = 4.804, *P* = 0.009 |
|  | UKMAC8 | Student’s T | T(4) = 6.685, *P* = 0.003 |
| UKMAC7 | UKMAC8 | Student’s T | T(4) = 4.934, *P* = 0.008 |
| (d) Cytopathic effect (10^6^ trophozoites; 6-hour co-incubation) | | | |
| UKMAC4 | AC20 | Student’s T | T(4) = 12.937, *P* < 0.0001 |
|  | UKMAC7 | Student’s T | T(4) = 9.744, *P* = 0.001 |
|  | UKMAC8 | Student’s T | T(4) = 8.455, *P* = 0.001 |
| UKMAC1 | AC20 | Student’s T | T(4) = 13.427, *P* < 0.0001 |
|  | UKMAC7 | Student’s T | T(4) = 9.606, *P* = 0.001 |
|  | UKMAC8 | Student’s T | T(4) = 8.223, *P* = 0.001 |
| AC20 | UKMAC7 | Student’s T | T(4) = 3.354, *P* = 0.028 |
|  | UKMAC8 | Student’s T | T(4) = 8.245, *P* = 0.001 |
| UKMAC7 | UKMAC8 | Student’s T | T(4) = 2.915, *P* = 0.043 |
| (f) Growth rate of trophozoites | | |  |
| UKMAC4 | UKMAC1 | Student’s T | T(4) = 7.182, *P* = 0.002 |
|  | AC20 | Student’s T | T(4) = 21.077, *P* < 0.0001 |
|  | UKMAC7 | Student’s T | T(4) = 4.706, *P* = 0.009 |
| UKMAC1 | AC20 | Student’s T | T(4) = 21.021, *P* < 0.0001 |
|  | UKMAC7 | Student’s T | T(4) = 2.833, *P* = 0.047 |
| AC20 | UKMAC7 | Student’s T | T(4) = 21.320, *P* < 0.0001 |
|  | UKMAC8 | Student’s T | T(4) = 13.153, *P* < 0.0001 |
| (g) MBP gene expression | | |  |
| UKMAC4 | UKMAC1 | Student’s T | T(4) = 31.698, *P* < 0.0001 |
|  | AC20 | Student’s T | T(4) = 61.707, *P* < 0.0001 |
|  | UKMAC7 | Student’s T | T(4) = 11.637, *P* < 0.0001 |
|  | UKMAC8 | Student’s T | T(4) = 14.605, *P* < 0.0001 |
| UKMAC1 | AC20 | Student’s T | T(4) = 33.956, *P* < 0.0001 |
|  | UKMAC7 | Student’s T | T(4) = 6.193, *P* = 0.003 |
|  | UKMAC8 | Student’s T | T(4) = 4.545, *P* = 0.010 |
| AC20 | UKMAC7 | Student’s T | T(4) = 3.923, *P* = 0.017 |
|  | UKMAC8 | Student’s T | T(4) = 12.906, *P* < 0.0001 |
| UKMAC7 | UKMAC8 | Student’s T | T(4) = 3.246, *P* = 0.032 |
| (h) AhLBP gene expression | | |  |
| UKMAC4 | AC20 | Independent T | T(4) = 3.211, *P* = 0.033 |
|  | UKMAC7 | Independent T | T(4) = 7.131, *P* = 0.002 |
|  | UKMAC8 | Mann-Whitney U | Z = 1.993, *P* = 0.046 |
| AC20 | UKMAC8 | Mann-Whitney U | Z = 1.993, *P* = 0.046 |
| UKMAC7 | UKMAC8 | Mann-Whitney U | Z = 1.993, *P* = 0.046 |
